# Imaging findings of right colon diverticulitis that mimics colon cancer: a case report

**DOI:** 10.4076/1757-1626-2-6289

**Published:** 2009-07-17

**Authors:** Lamprini K Kosma, Panagiota N Galani, Sofia P Lafoyianni

**Affiliations:** Radiology Department, A Fleming General Hospital14, 25^th^ Martiou Street, 15127 Melissia, AthensGreece

## Abstract

We present a case report of a patient with imaging findings of right colon diverticulitis that mimics colon cancer. The computed tomography showed a segment of narrowing with shoulder formation at ascending colon suggesting cancer. The colonoscopy and the follow-up imaging clarify the diagnosis of diverticulitis. We assess the value of computed tomography findings of acute diverticulitis in excluding cancer.

## Introduction

Clinically right-sided diverticulitis represents one of the greatest mimic of acute appendicitis. At the same time, clinically colon cancer may mimics diverticulitis. With current thin-section helical CT, most healthy appendixes can be revealed and the final aim is to rule out colon cancer.

To the best of our knowledge, no specific CT criteria have been established, especially for right-sided colonic diverticulitis and there is an overlap at least of 10% in the imaging appearance of these two conditions. We describe here a case of right colon diverticulitis that mimics colon cancer. We reviewed the current literature and we present the most common conventional CT findings for each situation.

## Case presentation

**Figure 1.**
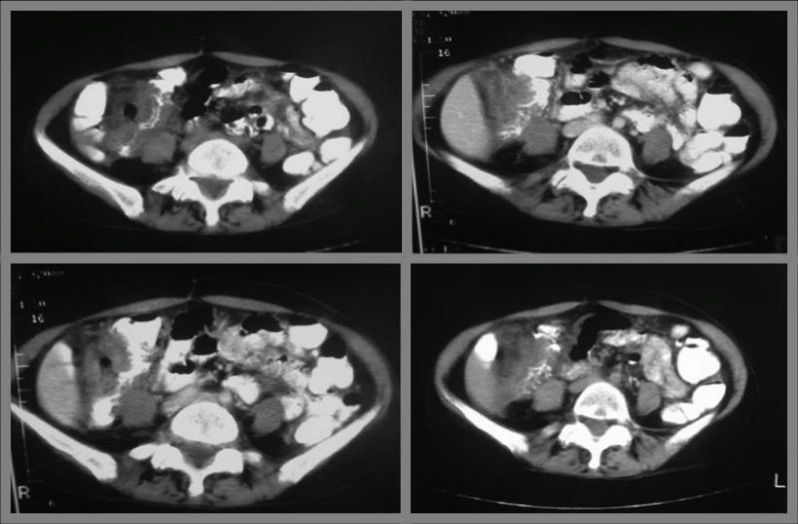
Non-contrast **(Top left)** and contrast **(Top right, Bottom left, Bottom right)** enhanced abdominal CT scan shows a segment of narrowing with shoulder formation of ascending colon and minimal inflammatory changes of adjacent fat.

A 60-year-old Caucasian Greek woman was referred to our hospital with a mild pain in the right lower quadrant for 5 days and axillary body temperature 38°C. Laboratory tests were within normal range. Abdominal examination showed extensive tenderness without significant rebound and a palpable mass. An abdominal contrast enhanced CT scan showed a short segment of narrowing with shoulder formation of ascending colon and minimal inflammatory changes in adjacent fat. The air into the thickened wall suggested being local perforation (Figure 1). A colonoscopy examination was obtained for further investigation. Colonoscopy showed thickening of a segment of ascending colon wall, a simple inflamed diverticula and contained rupture.

**Figure 2.**
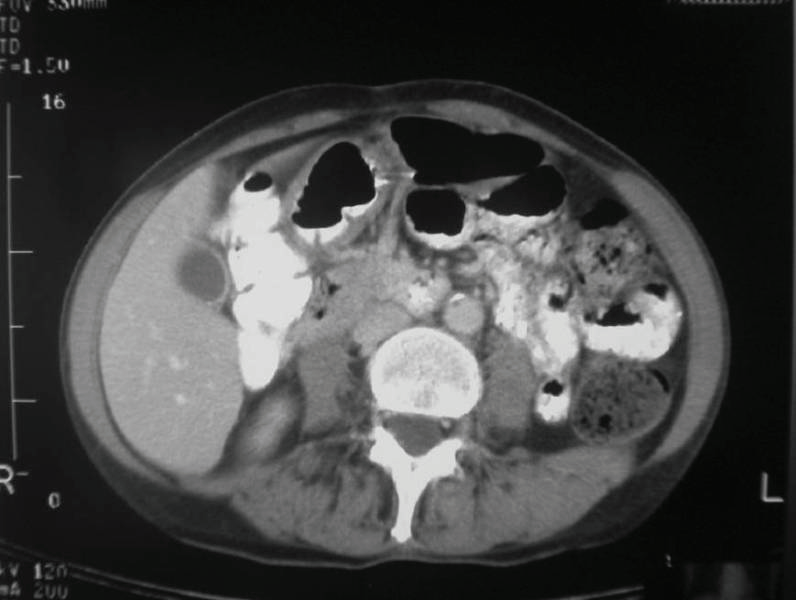
Follow up abdominal CT scan of the same patient after one month. There aren’t any pathologic findings.

The one month follow-up abdominal CT scan revealed no pathology and the diagnosis of diverticulitis was confirmed (Figure 2).

## Discussion

10 to 35 percent of patients with diverticula anywhere in the colon subsequently develop diverticulitis. Right side colonic diverticulitis is considered to be a rare condition in the Western population, but occurs with greater frequency in Asians. Only 1-5% of diverticula occur in the right colon, when 95% occur in left colon [1]. Recently the use of CT in patients with abdominal pain has demonstrated that acute diverticulitis of cecum and ascending colon is more common and has to receive more attention.

Diverticulitis is a benign and primarily nonsurgical disease that must be differentiated preoperatively from colonic carcinoma. Several groups of researchers have reported that accurate distinction between these two diseases is not always possible with CT findings and further investigation should be performed. On the other hand, some studies have suggested helpful CT findings of diverticulitis that can exclude carcinoma.

The two CT findings of right-sided colonic diverticulitis that most distinguishes it from colonic carcinoma are the inflamed diverticula and the preservation of an enhanced pattern of the involved colonic wall [2]. The recognition of inflamed diverticula is important, happens in 85-97% of patients and usually located at the level of maximal pericolic inflammation and maximal wall thickening. Inflamed diverticula, when defined on CT as diverticula, is associated with a thickened, enhancing diverticular wall and peridiverticular inflammatory changes, showing various levels of attenuation depending on their contents: fecalith, fecal material, air, fluid or soft-tissue attenuation [3].

Thin-section helical CT with intravenous contrast can certainly reveal the wall enhancement pattern that represents a very common finding. The above mentioned pattern is referred to an inner hyperattenuation layer, thickened middle layer of low attenuation and an outer hyperattenuation layer. We have to keep in mind that any other disease resulting in submucosal or muscular hypertrophy (e.g. ischemic or inflammatory bowel disease) can reproduce this finding.

However, it could be useful in differentiation especially from right-sided colonic carcinoma, because the axis of the right-sided colon is perpendicular to the CT plane and shouldering of colonic carcinoma (which can be helpful in excluding benign wall thickening) is rarely seen in right-sided colon [4].

In some cases of diverticulitis, contrast materials collect in an arrowhead shape as the inflammation spreads through the colonic wall. The appearance and frequency of occurrence of the “arrowhead sign” at CT depend on the degree of colonic luminal distention, the orientation of the affected bowel relative to the axial scanning plane and the amount of potentially obscurant adjacent inflammation. In addition to contrast material, luminal air may also accumulate at the site of focal wall thickening, resulting in an “air arrowhead sign”. In a relative study, this sign was the specific CT finding of diverticulitis (100% specificity). It was excluded the location of the anatomic cecal apex (1-4 cm below the ileocecal valve) where “arrowhead sign” were considered specific for appendicitis [5].

Wall thickness of the involved colon was markedly variable. Inflammation first leads to asymmetric thickening in the area of pericolic inflammation and later causes circumferential thickening in advanced stages. It is well known also that wall thickening suggests neoplasms [6]. Thickening of the colonic wall between 1 and 3 cm is reported to be one of the pitfalls in the CT diagnosis of diverticulitis [7]. Eccentric wall thickening and circumferential thickening with shoulder formation are atypical findings for diverticulitis, when luminal mass has high sensitivity for colon cancer.

According to Chintapalli et al, segment evolvement greater than 10 cm supports the diagnosis of diverticulitis, when length less than 5 cm is almost diagnostic for cancer. At the same time the degree of pericolic inflammation favors the diagnosis of right colon diverticulitis. The presence of pericolonic edema has been detected more often in diverticulitis but the numbers were not large enough in this study to attain statistical significance [8].

A pericolonic soft-tissue mass inseparable from the wall of the colon refers to a pericolonic abscess or phlegmon [9]. Current literature refers only a case of a patient where thrombosis involves the superior mesenteric vein as a complication of diverticulitis [2]. Chintapalli et al conclude that fluid at the root of mesentery and extraluminal air or fluid have sensitivities for diverticulitis of 50% and 30%, respectively. Additionally, the presence of lymph nodes has sensitivity for colon cancer 63% [8].

In general, there is no difference in the treatment of right side diverticulitis compared to left side diverticulitis. As most cases remain clinically unimminent, surgery is indicated only in complicated right sided cases [10].

## Conclusions

Careful interpretation of CT findings that takes into account all the aforementioned criteria could lead to an unequivocal and accurate diagnosis in about 50% of right sided colonic diverticulitis. Cases with less confident diagnosis need further work up including endoscopy and biopsy.

## Abbreviation

CT: Computer tomography
